# Odor Modification in Rigid Polyethylene Waste: Impact of Different Washing on Sensory Analysis and Characterization

**DOI:** 10.1002/gch2.70114

**Published:** 2026-05-29

**Authors:** Tiago G. A. Belé, Martijn Roosen, Helene M. Loos, Steven De Meester, Andrea Buettner

**Affiliations:** ^1^ Friedrich‐Alexander‐Universität Erlangen‐Nürnberg (FAU) Chair of Aroma and Smell Research Department of Chemistry and Pharmacy Erlangen Germany; ^2^ Laboratory for Circular Process Engineering (LCPE), Department of Green Chemistry and Technology, Faculty of Bioscience Engineering Ghent University – Campus Kortrijk Kortrijk Belgium; ^3^ Fraunhofer Institute for Process Engineering and Packaging IVV Freising Germany

**Keywords:** 2D‐GC‐MS/O, circular economy, cOEDA, deodorization, gas chromatography, plastics recycling, sensory analysis

## Abstract

Persistent odor in recycled polyethylene (PE) limits its use in high‐demand applications, necessitating effective deodorization. We investigated how washing affects the odor profile and intensity of post‐consumer rigid PE, using sensory analysis, comparative odor extract dilution analysis (cOEDA), and two‐dimensional gas chromatography‐mass spectrometry/olfactometry (2D‐GC‐MS/O). Three methods were evaluated, varying in temperature and surfactant use: room temperature water (RT, 25°C), hot water (HW, 80°C), and detergent with caustic soda (DW). Sensory analysis showed that no treatment significantly reduced overall odor intensity or hedonic ratings, although washing modified the qualitative odor profile. Median values indicated a modest downward trend for DW (17% reduction), though not statistically significant. cOEDA and 2D‐GC‐MS/O results aligned with these qualitative shifts, including reduced moldy and increased soapy and flowery notes. Of 32 odor‐active compounds detected in post‐consumer rigid PE, 28 were identified. Rose oxide, dihydromyrcenol, hexyl salicylate, and α‐hexylcinnamaldehyde are reported for the first time in post‐consumer PE. This study deepens understanding of post‐consumer PE odor and highlights challenges in developing advanced deodorization techniques for the circular economy.

## Graphical abstract

Post‐consumer rigid polyethylene waste was characterized for odor‐active compounds using sensory analysis, comparative odor extract dilution analysis, and 2D‐GC‐MS/O. Twenty‐eight odorants were identified, with four reported for the first time. A direct comparison with polypropylene suggests polymer‐specific adsorption–desorption dynamics governed by crystallinity and surface energy, informing targeted deodorization strategies for polyolefin recycling.

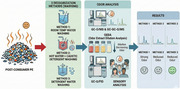

**Abbreviations**
2D‐GC‐MS/O: two‐dimensional gas chromatography‐mass spectrometry/olfactometry
cOEDA: comparative odor extract dilution analysis
DW: detergent washed samples
FID: flame ionization detector
GC‐MS: gas chromatography‐mass spectrometry
GC‐O: gas chromatography with olfactometry
HDPE: high‐density polyethylene
HW: caustic hot water washed samples, with water at 80°C
OD: odor dilution.
PE: polyethylene
RT: room temperature washed samples with water at 25°C
UW: unwashed samples
VOC: volatile organic compound

## 1 Introduction

With an increased awareness of the plastic industry's contribution to environmental problems, the European Union has been developing policies to transition plastics from being used as a linear to a circular material. For example, the European Union Directive 2018/852 on Packaging and Packaging Waste [1] sets higher recycling targets per material, as 50% for plastic packaging by 2025 and 55% by 2030. Moreover, in 2020 a Circular Economy Action Plan was launched by the EU to create a model of production and consumption in which reusing, repairing, refurbishing, and recycling are key strategies [2].

Indeed, recycled plastics are used in a broad range of applications, yet, currently not always in a closed loop, connecting production to end‐of‐life point [3]. Recycling strategies are amongst the tools used to increase plastic circularity [4]. A significant challenge in recycling packaging is the persistent odor of the final recyclate, which often renders the recycled polymer unsuitable for use in high‐demand applications [5, 6].

With regards to PE, it is well known that this type of polymer can absorb and/or adsorb other chemicals [7], and potentially odorants [8], thus, being able to elicit undesirable smell. Such an issue has consequently become the interest of researchers and entrepreneurs alike, in an effort to deodorize plastics [9, 10]. In a study, Strangl et al. [6] evaluated a modified recycling method for HDPE to reduce odors in recycled pellets [6]. The method involved decontaminating the pellets in a thermostated reactor, which significantly reduced the overall odor intensity when comparing difference residence times. While such a strategy is innovative, it might prove itself financially unviable. The change of paradigm and technology upgrades are a challenge to overcome, and the recycling industry still relies heavily on washing methods as a means for deodorization.

More recently, López Martínez et al. [11] investigated the odor characterization of recycled HDPE from waste collection plants subjected to different washing and processing steps. Their results showed that a combination of acetone pre‐washing followed by extrusion and injection processing effectively removed volatile compounds responsible for undesirable odors, without compromising the mechanical properties of the recyclate. These findings underline the potential of solvent‐based pre‐treatments as a complementary strategy to conventional aqueous washing in polyolefin recycling.

Regarding PP, Saini et al. [12] compared three treatment approaches to reduce volatile organic compounds (VOCs) and odor from post‐consumer recycled PP: polyethylene glycol (PEG) extraction, heated air purging, and additivation with zeolites. Zeolite additivation achieved the highest VOC reduction (89%), followed by heated air purging (78%) and PEG extraction (73%), while none of the methods significantly altered the melt flow index. This study illustrates that, alongside PE, post‐consumer PP also demands targeted deodorization strategies, and that the efficacy of such treatments varies considerably depending on polymer type, contaminant profile, and the physicochemical interactions involved. Despite the growing interest in alternative deodorization technologies for both PE and PP, a direct side‐by‐side comparison of the odorant profiles and washing responses of these two major polyolefins from the same waste stream remains scarce in the literature.

Thus, to further contribute toward the optimization of plastic deodorization, with an emphasis on PE, the present study focuses on:

1. Odor profiling of rigid PE waste before and after three different washing conditions: (A) with water at room temperature (25°C), (B) applying hot water (80°C), and (C) a caustic solution with detergent (80°C).
2. Evaluating and identifying the odor‐active compounds in unwashed and washed plastics using comparative odor extract dilution analysis (cOEDA), and (two‐dimensional) gas chromatography‐mass spectrometry/olfactometry (2D‐GC‐MS/O).
3. Evaluating the washing efficiency regarding odor profile, overall intensity, and hedonic ratings from sensory analyses executed by a trained panel.
4. Comparing results and deodorization efficiency against PP in a report previously published [13].

## 2 Materials and Methods

### 2.1 Sample Description

A total amount of 10 kg of PE waste was sampled at a Belgian waste management company. The material consisted predominantly of high‐density polyethylene (HDPE) rigid packaging, mainly bottles and containers. Visual inspection of the sorted stream indicated that the feedstock comprised household packaging from mixed curbside collection, with prior‐use categories including food packaging (e.g. milk, juice, and cooking‐oil bottles), household cleaning agents, and personal‐care products. Minor contamination with labels, adhesive residues, and small amounts of non‐PE material (caps, closures) was present, as is typical for post‐consumer rigid PE fractions from materials recovery facilities. Plastic packaging products were sampled from the conveyor belt after sorting in an MRF (materials recovery facility). Afterward, plastics were shredded in the lab using a Piovan‐type RSP15/30 shredder with a sieve diameter of 8.0 mm to obtain more homogeneous samples and stored following the protocol described by [9] for two days.

### 2.2 Washing Procedures

Batch‐washing experiments were performed on unwashed (UW) PE waste. The plastic material was washed with different washing media in a 2 L round‐bottom flask. The tested washing media were water at 25°C (room temperature—in the following: RT), water at 80°C (hot washing—in the following: HW), and caustic soda at 80°C with water containing 2 m% NaOH and CTAB at a concentration of 9.2 mm (detergent washing—in the following: DW). A solid‐liquid ratio of 50 g plastic on 1 L washing medium was applied for each washing experiment. Plastics were stirred using an agitator at a speed of 200 rotations per minute. The washing medium was heated up to the desired temperature of 25°C or 80°C through a heating mantle. The washing time was 10 min for each washing procedure. Following washing, the plastics were rinsed with water at 25°C to eliminate any leftover detergents, and then placed in a desiccator to dry at room temperature for 24 h. Plastics were stored for a maximum of two weeks at 0°C before analysis [13].

### 2.3 Chemicals

Anhydrous sodium sulfate and dichloromethane (DCM) were obtained from Th. Geyer, Renningen, Germany, and liquid nitrogen was purchased from Linde GmbH, Pullach, Germany. DCM was freshly distilled prior to use. A series of alkanes from n‐hexane to n‐triacontane (Fluka, Steinheim, Germany, and Sigma–Aldrich, Steinheim, Germany) was used to determine linear retention indices (RIs). All reference compounds used for identification of odorants were purchased from the same suppliers listed in Belé et al. [13], with the following additional references: 1‐methyl‐β‐ionone ≥ 99%; 3‐methylbutanoic acid ≥ 99%; (E,E)‐nona‐2,4‐dienal ≥ 85%; 6‐methyl‐5‐hepten‐2‐one ≥ 90%; butanoic acid ≥ 99.5%; nonanal ≥ 95%; α‐pinene ≥ 97%.

### 2.4 Sensory Evaluation

#### 2.4.1 Trained Sensory Panel

Panelists were trained assessors from the Chair of Aroma and Smell Research (Department of Chemistry and Pharmacy—FAU, Erlangen, Germany). Five female and three male panelists (*n* = 8; age range: 23–35 years) participated in the sensory evaluation of all plastic samples. Before participation in the experiments, panelists were trained during weekly sessions with selected odor solutions to identify and name odorants correctly, following the general principles of ISO 8586 [14]. The same eight panelists evaluated all four treatment conditions (UW, RT, HW, DW) in a within‐subject design, to ensure paired comparisons across all groups.

#### 2.4.2 Procedure

The sensory evaluation was carried out in a dedicated sensory room located at the Chair of Aroma and Smell Research (FAU, Erlangen, Germany). For the descriptive evaluation together with odor intensity ratings, 15 g (±0.1) of the sample were presented to the panel inside a 140 mL covered glass vessel. For each sample, a three‐digit random code was assigned and the order of presentation was randomized across panelists to minimize order effects; panelists were not informed of the treatment identity (blinded design). The odor profile analyses were carried out in three phases according to instructions provided in ISO 13299 [15]. In the first phase, the sensory impressions were individually described by the panelists. After collecting all odor qualities, the panel agreed on the main attributes as well as on the respective odorant references, with approval from more than half of the panelists. Aqueous solutions of fruity, citric, soapy, and flowery smells were prepared with ethyl 2‐methylbutanoate, nonanal, decanal, and linalool, respectively. Actual pieces of cardboard paper were used as a reference for the odor attribute cardboard‐like [16], as panelists could not agree on a reference for the moldy smell. In the last phase, the panelists were asked to evaluate the intensities of these attributes, in parallel with the overall intensities of the samples on a scale from 0 (no perception) to 10 (strong perception). Additionally, the hedonic perception of the samples was evaluated on a unipolar scale from 0 (dislike) to 10 (like). This 0–10 unipolar scale was selected because the aim of the study was to compare the relative liking of plastic smell rather than to classify them as pleasant or unpleasant, where panelists were instructed that 0 was pointed as very unpleasant, 5 neutral, and 10 very pleasant, as according to Cabanes et al. [17]. Results as medians were plotted in a spider‐web diagram. No formal outlier‐removal procedure was applied; all individual ratings were retained in the analysis to preserve the full variability of the small panel.

#### 2.4.3 Data Analysis

To evaluate differences between plastic treated with the different washing processes with respect to intensity and hedonic ratings, statistical analysis was done using SPSS (IBM, New York, USA) and Microsoft Excel software (Microsoft, Redmond, USA), applying the Kruskal‐Wallis test and Dunn‐Bonferroni post‐hoc tests (pairwise comparisons with significance values adjusted by Bonferroni correction). All eight panelists assessed every treatment condition, yielding N = 32 total observations (*n* = 8 per group). The same panelists evaluated all four conditions, providing a within‐subject structure that reduces inter‐individual variability. It should be acknowledged that with *n* = 8 per group, the statistical power to detect small‐to‐moderate effect sizes is limited, and non‐significant results should therefore be interpreted as absence of evidence for a difference rather than evidence of equivalence. Effect sizes are discussed qualitatively alongside p‐values in the Results section to provide a more complete picture of the practical significance of any observed trends.

### 2.5 Isolation of Volatiles

To extract volatile organic compounds (VOCs), 10 g (± 0.1 g) of shredded plastic was stirred in 250 mL of DCM for 30 min. After filtering, the volatile portion was separated from non‐volatile compounds using the Solvent Assisted Flavor Evaporation (SAFE) method as described by Engel and colleagues [18]. The distillation was carried out under high vacuum with the flask in a 50°C water bath and the SAFE apparatus set to 55°C. The distillate, collected into a liquid nitrogen‐cooled flask, was thawed at room temperature and dried over anhydrous sodium sulfate. After filtration, DCM was evaporated by Vigreux distillation to reduce the volume to 3 mL, then further concentrated to 0.1 mL via micro‐distillation.

### 2.6 Gas Chromatography‐Olfactometry (GC‐O), Odor Extract Dilution Analysis, and Two‐Dimensional GC‐MS/O

GC‐O analysis was carried out using a 2D gas chromatography system with mass spectrometric and olfactometric detection (2D‐GC‐MS/O), employing two Agilent 7890B systems (Agilent Technologies, Santa Clara, USA) coupled via a CTS 1 cryogenic trapping device (Gerstel GmbH & Co. KG). The second chromatographic dimension was interfaced with an Agilent 5977 Quadrupole MS. A 2 µL aliquot of each distillate was introduced using a Multipurpose Sampler MPS 2XL (Gerstel GmbH & Co. KG) in cold on‐column mode at an oven start temperature of 40°C. For separation, a DB‐FFAP column (30 m × 0.32 mm, 0.25 µm film; J&W Scientific, Agilent Technologies GmbH) was employed in the first dimension and a DB‐5 column (30 m × 0.25 mm, 0.25 µm film; J&W Scientific, Agilent Technologies GmbH) in the second. Both ovens followed the same programme: an initial hold at 40°C for 2 min, followed by a ramp of 8 °C/min up to the final temperature (240°C, 10 min for DB‐FFAP; 250°C, 5 min for DB‐5). The effluent from the first column was divided between an olfactory detection port (ODP, 270 °C) and a flame ionisation detector (FID, 250°C). Helium was used as carrier gas at 2.5 mL/min. Defined retention windows (∼0.4 min) were trapped at −100°C in a CTS system via the MCS2 multi‐column switching device (Gerstel GmbH & Co. KG). After thermal desorption at 250°C, analytes were transferred to the second GC. At the outlet of the DB‐5 column, the flow was split between the ODP (270°C) and the MS. Mass spectra were acquired in EI mode at 70 eV over an m/z range of 35–400. Odor extract dilution analysis (OEDA) was performed to determine odor dilution (OD) factors using stepwise distillate dilution with DCM (1:2 v/v) on the FFAP column. GC‐O analyses used undiluted distillate (OD factor 1) and serial dilutions with OD factors of 3, 9, 27, 81, 243, 729, 2187, and 6561. Each compound's OD factor was determined as the highest dilution where its odor remained detectable at the sniffing port. As OD 1 was too heavily loaded with odor impressions, the GC‐O analyses were started at OD 3. The odor‐active areas at OD 3 were additionally evaluated by a second trained person to ensure the detection of all odorants. For each treatment condition (UW, RT, HW, DW), a single SAFE distillate was prepared and subjected to the full cOEDA dilution series; the reproducibility of individual OD factors was therefore not assessed across independent replicates. This analytical design is consistent with previous cOEDA studies on recycled plastics [13, 17] and is appropriate for a screening‐level comparison, although it means that compound‐level OD factors should be interpreted semi‐quantitatively.

### 2.7 Identification Criteria of Odorants

For preliminary identification, the retention index (RI) and odor characteristics (O) of odor‐active compounds detected during GC‐O analyses with DB‐FFAP and DB‐5 capillary columns were compared to those of reference compounds (RC), which were selected from an internal odorant database. Subsequently, 2D‐GC–MS/O analysis was conducted to determine each molecular structure by matching the mass spectrum (MS) of the odorant with that of the corresponding RC. When a match was achieved on all criteria—RI, O, and MS—the odorant was considered positively identified. In Table 1, the identification criteria column indicates which data supported each assignment: RI (retention index match on both columns), OQ (odor quality match), MS (mass spectrum match), and RC (co‐injection with authentic reference compound). Compounds for which MS data could not be obtained or for which no authentic standard was available are listed as tentatively identified or unknown, respectively.

**TABLE 1:** Odor‐active compounds identified in distillates obtained from shredded post‐consumer rigid PE before (UW) and after washing processes (RT, HW, DW). Displayed are the odor qualities, retention indices (R.I.), and OD factors, as well as the respective identification criteria.

		R.I.		OD‐factor				
Compound	Odor quality	DB‐FFAP	DB‐5	UW	RT	HW	DW	Identification criteria
α‐pinene	resin‐like, conifer‐like	1055	887	27	27	27	27	RI, OQ, MS, RC
ethyl 2‐methylbutanoate	fruity, strawberry‐like	1108	860	729	243	243	243	RI, OQ, MS, RC
propyl 2‐methylbutanoate	fruity, pineapple‐like	1138	948	243	243	243	243	RI, OQ, MS, RC
1,8‐cineole	eucalyptus‐like	1185	1019	27	27	27	27	RI, OQ, MS, RC
1‐octen‐3‐one	mushroom‐like	1291	985	27	27	27	27	RI, OQ, RC
rose oxide	soapy, flowery	1333	1110	81	81	81	81	RI, OQ, MS, RC
6‐methyl 5‐hepten‐2‐one	fruity	1354	950	27	27	27	27	RI, OQ, MS, RC
nonanal	soapy, citrus‐like	1392	1105	27	27	27	27	RI, OQ, RC
tetrahydrolinalool	flowery, soapy	1423	1098	81	81	81	81	RI, OQ, MS, RC
dihydromyrcenol	soapy, citrus‐like, flowery	1465	1062	27	27	27	27	RI, OQ, MS, RC
linalool	flowery	1545	1097	243	243	243	81	RI, OQ, MS, RC
butanoic acid	cheesy, sweaty	1620	815	9	9	9	9	RI, OQ, RC
unknown	flowery	1675	n.d.	27	27	9	9	
(*E*,*E*)‐nona‐2,4‐dienal	fatty	1692	1208	9	9	9	3	RI, OQ, RC
unknown	rubber‐like, plastic‐like	1700	n.d.	3	3	3	3	
3‐methylbutanoic acid	cheesy	1710	921	3	3	3	3	RI, OQ, RC
α‐damascone	fruity, apple‐like	1750	1385	81	81	81	81	RI, OQ, MS, RC
unknown	leather‐like, corky	1800	n.d.	27	27	27	27	
unknown	flowery	1809	n.d.	27	27	27	27	
α‐isomethylionone	flowery	1850	1489	729	243	729	81	RI, OQ, MS, RC
verdyl acetate	fruity, banana‐like	1888	1432	243	243	729	81	RI, OQ, MS, RC
β‐ionone	violet‐like, flowery	1918	1494	2187	729	27	81	RI, OQ, MS, RC
1‐methyl‐β‐ionone	flowery	1970	n.d.	27	27	729	27	RI, OQ
(*trans*)‐4,5‐epoxy‐(*E*)‐dec‐2‐enal	metallic	2000	1380	27	27	27	243	RI, OQ, RC
methylcyclomyrcetone isomer	flowery	2100	1698	2187	27	27	729	RI, OQ, MS, RC
eugenol	clove‐like	2156	1361	2187	729	9	9	RI, OQ, MS, RC
2‐methoxynaphthalene	glue‐like, almond‐like	2185	1463	27	27	729	27	RI, OQ, MS, RC
hexyl salicylate	soapy	2200	1686	729	729	27	729	RI, OQ, MS, RC
decanoic acid	soapy	2277	1370	9	9	3	9	RI, OQ, RC
α‐hexylcinnamaldehyde	soapy, flowery	2366	1757	9	9	9	9	RI, OQ, MS, RC
vanillin	vanilla‐like	2575	1401	9	9	9	3	RI, OQ, RC
3‐phenylpropanoic acid	honeycomb‐like	2600	1338	9	9	9	3	RI, OQ, RC

RI: retention index according to Van den Dool and Kratz (1963), OD: Odor dilution factor on capillary DB‐FFAP according to Grosch (2001), MS: mass spectrum, n.d.: not detected, O: odor quality perceived at the ODP, RC: comparison of the respective data with a reference compound. Only compounds with OD factors equal or higher than 3 are depicted.

## 3 Results

### 3.1 Sensory Evaluation

#### 3.1.1 Hedonic Ratings, Intensity Ratings, and Odor Profiles

The hedonic rating of PE showed no significant differences between the groups (UW, RT, HW, DW), (H = 4.88, p = 0.181).

**FIGURE 1:**
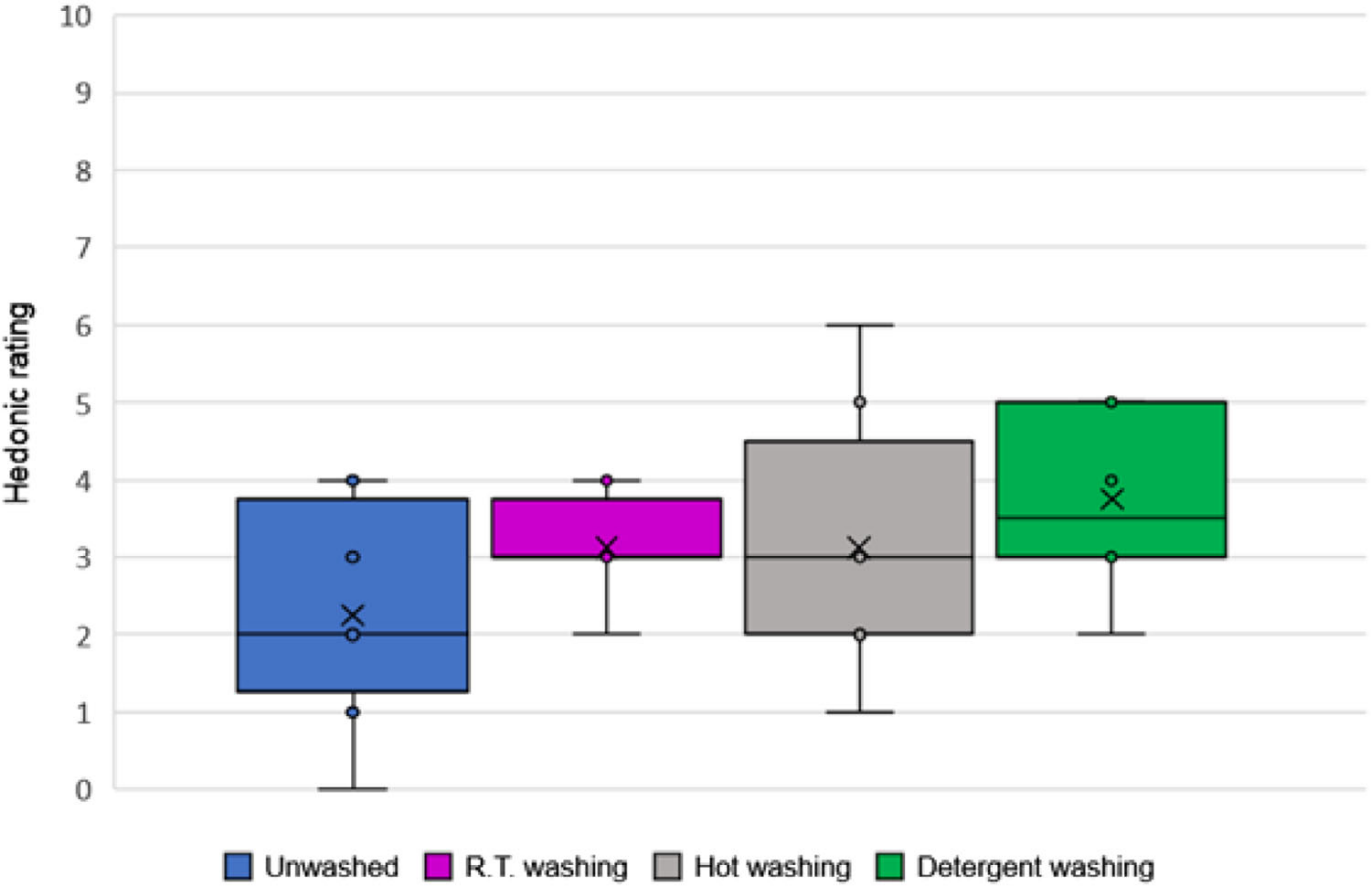
Overall hedonic ratings of PE before (UW) and after different washing processes (RT, HW, DW). Data were obtained from a panel of 8 participants. The scale is from 0 (dislike) to 10 (like). Boxplot data: lower whisker: minimum, higher whisker: maximum; dots: outliers; x: mean, dash: medians.

The lowest hedonic rating was obtained for the unwashed samples (Figure 1).

The median hedonic ratings for the PE samples treated with various washing methods revealed subtle differences, although the statistical tests did not show significant variations (p = 0.181). UW PE samples exhibited the lowest median rating of 2. The DW PE samples had the highest median rating of 3.5, suggesting a possible trend toward improved perception, though this was not statistically supported. Both RT and HW washing resulted in median ratings of 3, showing similar levels of sensory acceptability. Given the panel size of *n* = 8, these comparisons may be underpowered to detect small‐to‐moderate effects; the absence of significance should thus be interpreted cautiously.

**FIGURE 2:**
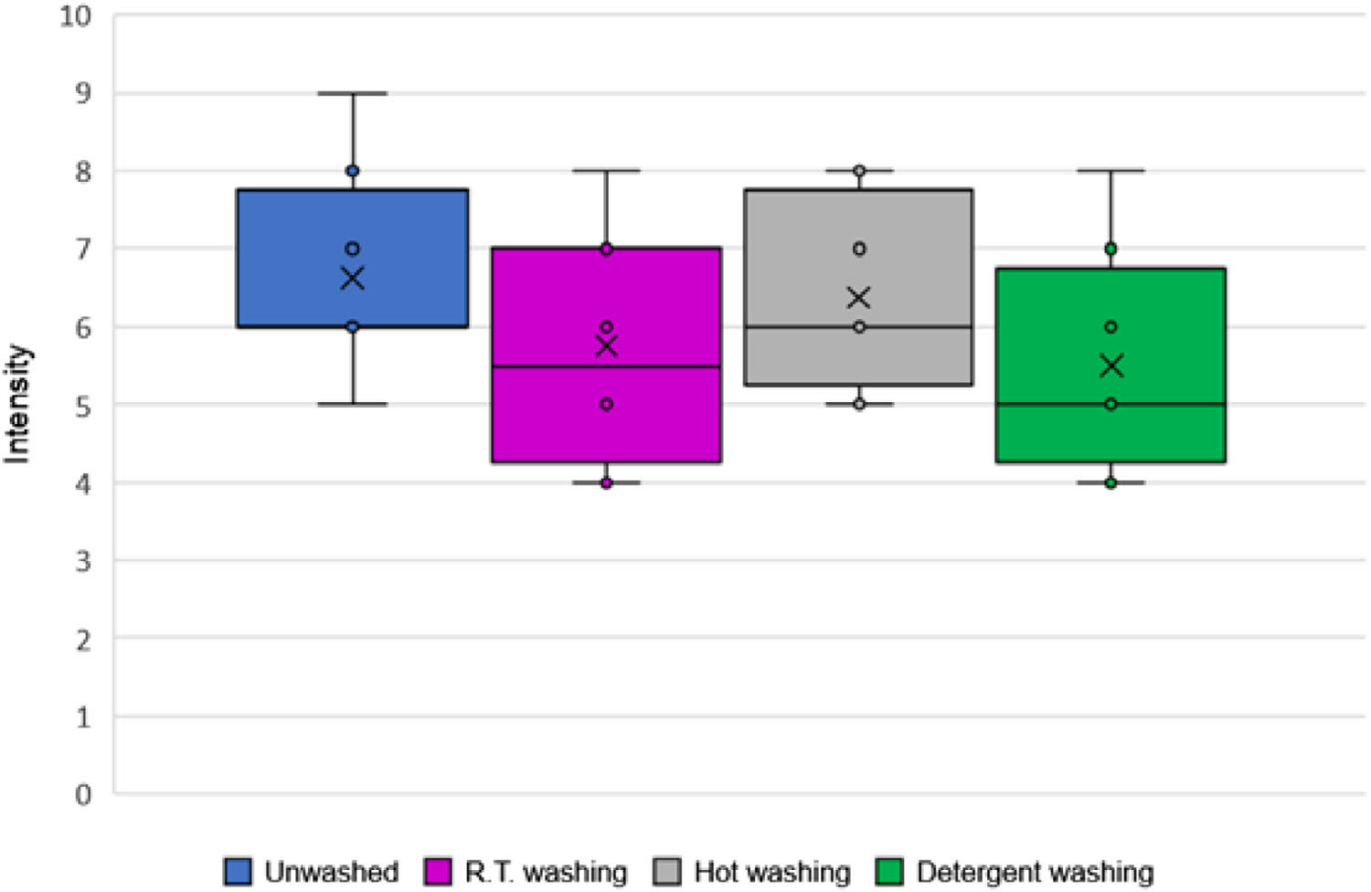
Overall odor intensities of PE before (UW) and after different washing processes (RT, HW, DW). Data were obtained from a panel of 8 participants. Scale is from 0 (no perception) to 10 (strong perception). Boxplot data: lower whisker: minimum, higher whisker: maximum; dots: outliers; x: mean, dash: medians.

Intensity ratings for PE are depicted in Figure 2. The Kruskal‐Wallis test for the intensity ratings of PE showed no significant differences between the groups (H = 3.64, p = 0.303).

The UW and HW PE samples both had the highest median rating of 6, indicating that heat alone, in this context, does not provide significant odor reduction compared to untreated plastic. RT washing resulted in a slightly lower median intensity of 5.5, while DW had the lowest median rating of 5, reflecting a modest reduction in perceived intensity.

**FIGURE 3:**
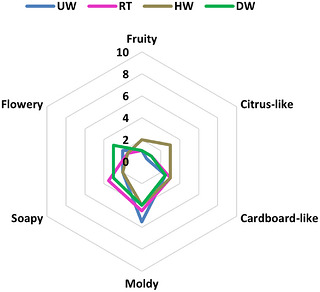
Odor profiles of PE before (UW) and after different washing processes (RT, HW, DW). Data are displayed as medians of intensity ratings from a panel of 8 participants. Scale is from 0 (no perception) to 10 (strong perception).

The odor profile analysis described the samples with the attributes fruity, citrus‐like, cardboard‐like, moldy, soapy, and flowery. The median intensity ratings determined for these attributes during the comparative odor profile analyses for PE are displayed in a spider web diagram (Figure 3). The moldy smell was the most intense smell in UW PE with a median intensity rating of 5.5.

Between washing methods, a slight change in the odor profile of rigid PE was observed. The moldy smell remained the most intense in the UW samples, with a median of 5.5, while the intensity decreased to 4.5 in the RT samples. In the HW and DW samples, the moldy smell was rated with a median intensity of 4. The soapy attribute also showed a higher intensity in the RT samples, with a median of 3.5, compared to 2 in the UW samples. Other attributes showed relatively higher intensities, notably citrus‐like (3) and fruity (2) in the HW samples, while in the DW samples, the soapy and flowery attributes both reached a median intensity of 3.

The statistical analysis for odor profile analysis of PE samples after washing treatments revealed no significant differences for most attributes (*p* > 0.05). For the fruity attribute, the Kruskal‐Wallis test showed no significant effect of the washing treatments (H = 3.66, p = 0.301). Similarly, no statistically significant differences between UW, RT, HW, and DW groups were found for the citric attribute (H = 6.35, p = 0.096). The cardboard attribute also showed no significant differences across treatments (H = 1.05, p = 0.788), nor did the soapy impression (H = 3.51, p = 0.320). The moldy note approached statistical significance (H = 7.63, p = 0.054), but post‐hoc pairwise comparisons did not reveal significant differences between specific groups after applying the Bonferroni correction (*p* > 0.05). Given the small panel size (*n* = 8 per group), this borderline result may reflect insufficient statistical power to detect a real effect rather than true equivalence. Finally, the different treatments showed no significant differences for the flowery attribute (H = 2.95, p = 0.399).

### 3.2 Characterization of the Odorants

Analysis of the diluted distillates of PE samples using GC‐O, utilizing a DB‐FFAP capillary, led to the detection of 32 odor‐active compounds (Table 1). The OEDA of UW post‐consumer rigid PE revealed six odorous substances with high OD factors between 729 and 2187. With an OD factor of 2187, the most pronounced compounds were β‐ionone (violet‐like, flowery), a methylcyclomyrcetone isomer (flowery), and eugenol (clove‐like). OD factors of 729 were determined for the compounds ethyl 2‐methylbutanoate (fruity, strawberry‐like), α‐isomethylionone (flowery), and hexyl salicylate (soapy).

Overall, 28 odorous compounds were identified based on chromatographic and mass spectrometric data and their smell characteristics (Table 1). These were, in addition to the six compounds with the highest OD factors, α‐pinene (resin‐like, conifer‐like), propyl 2‐methylbutanoate (fruity, pineapple‐like), 1,8‐cineole (eucalyptus‐like), 1‐octen‐3‐one (mushroom‐like), rose oxide (soapy, flowery), 6‐methyl‐5‐hepten‐2‐one (fruity), nonanal (soapy, citrus‐like), tetrahydrolinalool (flowery, soapy), dihydromyrcenol (soapy, citrus‐like, flowery), linalool (flowery), butanoic acid (cheesy, sweaty), (*E*,*E*)‐nona‐2,4‐dienal (fatty), 3‐methylbutanoic acid (cheesy), α‐damascone (fruity, apple‐like), verdyl acetate (fruity, banana‐like), 1‐methyl‐β‐ionone (flowery), (*trans*)‐4,5‐epoxy‐(*E*)‐dec‐2‐enal (metallic), 2‐methoxynaphthalene (glue‐like, almond‐like), decanoic acid (soapy), α‐hexylcinnamaldehyde (soapy, flowery), vanillin (vanilla‐like), 3‐phenylpropanoic acid (beeswax‐like, flowery, honey‐like).

**FIGURE 4:**
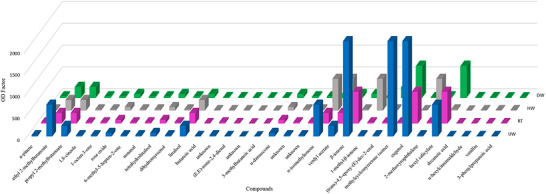
Overview of OD factors of compounds detected in post‐consumer PE before (UW) and after washing processes (RT, HW, DW).

The OD factors from the UW samples clearly showed some differences compared to those of the washed PE (Figure 4). Generally, the OD factors of the washed PE material were lower than those observed for the unwashed PE, exemplified by compounds such as ethyl 2‐methylbutanoate (fruity, strawberry‐like), α‐isomethylionone (flowery), and hexyl salicylate (flowery). The OD factor of ethyl 2‐methylbutanoate (fruity, strawberry‐like) decreased from 729 (UW) to 243 (RT, HW, DW), and α‐isomethylionone (flowery) decreased from 729 (UW) to 243 (RT) and 81 (DW). However, in a minority of cases, the OD factor did not change, or even increased, depending on the type of washing process. For example, an increase in OD factor was observed for 1‐methyl‐β‐ionone and 2‐methoxynaphthalene, with OD factors changing from 27 (UW, RT, DW) to 729 (HW).

## 4 Discussion

### 4.1 Odorant Composition and New Components Found

Several compounds, such as β‐ionone (flowery, violet‐like), eugenol (clove‐like), ethyl 2‐methylbutanoate (fruity, strawberry‐like), α‐isomethylionone (flowery), nonanal (soapy, citrus‐like), 1‐octen‐3‐one (mushroom‐like), (*E,E*)‐nona‐2,4‐dienal (fatty), and 3‐methylbutanoic acid (cheesy), have been described elsewhere as odorants originating from plastic waste, particularly in post‐consumer recyclates [6, 17, 19, 20]. These compounds are commonly reported in studies on recycled plastics due to their strong odor activity and persistence through washing and extrusion processes.

Nonetheless, and to the best of our knowledge, the compounds rose oxide, dihydromyrcenol, hexyl salicylate, and α‐hexylcinnamaldehyde are reported here for the first time in post‐consumer PE rigid plastics.

### 4.2 Potential Origin of the Components

The diverse odor‐active compounds identified in post‐consumer rigid PE suggest multiple contamination pathways beyond residues from detergent and cosmetic packaging. Many of the detected odorants, such as ethyl 2‐methylbutanoate (fruity, strawberry‐like) and β‐ionone (flowery, violet‐like), are commonly associated with food products and beverages such as juices and yoghurt [8, 21, 22], indicating that packaging for perishable goods may behave as an adsorbent for those compounds. The detection of eugenol (clove‐like) and vanillin (vanilla‐like), both known food additives [23, 24], reinforces this link to food‐contact materials such as rigid PE. In another instance, Czerny and Schieberle [25] demonstrated that aldehydes such as (*E*)‐2‐nonenal and (*E,Z*)‐2,6‐nonadienal exhibit a strong affinity for PE, being readily adsorbed from UHT‐milk into the polyethylene packaging [25]. The detection of aldehydes such as (*trans*)‐4,5‐epoxy‐(*E*)‐dec‐2‐enal (metallic) and (*E,E*)‐nona‐2,4‐dienal (fatty) in the present study is consistent with PE's known tendency to adsorb aldehydic compounds from food matrices. Although the affinity of aldehydes to PE was shown to be stronger than that of lactones and acids [25], the relatively low OD factors observed in the present study for these specific aldehyde‐type odorants suggest that their initial absorption during the product's service life may have been limited, or that washing was partly effective in their removal. Nevertheless, the detection of both food‐derived odorants (e.g., aldehydes, esters) and household/cosmetic‐related compounds in post‐consumer PE supports the notion of multiple, overlapping contamination pathways. The presence of these food‐associated compounds aligns with the established adsorptive capacity of PE packaging and highlights that food‐contact migration represents a relevant, albeit not always dominant, source of odor contamination in recycled rigid PE.

### 4.3 Washing Efficiency

The odor intensity of UW PE showed no significant difference from the odor intensity of the respective washed samples (RT, DW, HW). Although these values suggest that DW is the most effective in reducing odor intensity, the differences are minor, and the lack of statistical significance might imply a limited relevance for industrial applications. We hypothesize that when more aggressive washing such as HW and DW are performed, substances that are adsorbed onto the bulk of the plastic migrate to outer layers, thus increasing the odor perception.

In another study, researchers evaluated the washing of LDPE bags with hot water and showed a significant decrease in the overall intensity of the sample, from 8 to 6.3 (0–10 scale) [17]. In this current study, washing with hot water was not significant for PE with respect to intensity differences. This might be due the higher surface area of LDPE bags when compared to PE, thus, with LDPE being subjected to a better removal performance. Since many of the detected odorants, such as eugenol, linalool, and vanillin, contain polar hydroxyl or methoxy groups, the nonpolar polymer matrix interacts with these compounds primarily through weak van der Waals forces rather than specific polar interactions, which can present limitations to the ability of water‐based washing agents to effectively extract them from the polymer bulk.

It is noteworthy, however, to address the qualitative change in the odor profile of PE. The highest intensity was obtained in UW PE with respect to the moldy smell, whereas the HW PE most intense smell was rated as citrus‐like, and that of DW PE as flowery. It is possible that the higher intensity of the moldy percept in the UW samples is due to a synergistic effect of different odors in the sample. These observations suggest a trend in which DW washing may shift the odor character of the plastic toward more acceptable attributes from a sensory perspective, though the overall intensity was not significantly reduced and this interpretation should be considered tentative given the limited panel size.

The UW and HW PE samples both had the highest median rating of 6, indicating that heat alone, in this context, does not provide significant odor reduction compared to not washing plastic. This also indicates that, while such methods improve the pleasantness of odor perception, it does not correlate with odor reduction when comparing UW and HW for PE.

Roosen et al. [9] concluded that using only detergent, without NaOH, is a better deodorization strategy for rigid PE than a washing step with caustic soda and detergent [9]. This current report adds to the current knowledge as it includes a comprehensive sensory analysis and cOEDA, which is paramount, since a material's odor may not align with the most abundant substances in the sample or reflect true deodorization, as many VOC's can be odorless. In this study, none of the washing methods significantly reduced the overall odor intensity of the samples, as supported by the sensory panel results.

### 4.4 Research Implications and Comparison Between Polymers

Table 2 provides a side‐by‐side comparison of the PE (present study) and PP [13] datasets. Both polymers were sourced from the same Belgian MRF, processed under identical washing conditions (RT, HW, DW), and evaluated by the same trained panel (*n* = 8). Key differences include: (i) the PP companion study found a statistically significant improvement in hedonic rating for DW (median 2 → 5, p = 0.001), whereas PE showed only a non‐significant trend (median 2 → 3.5, p = 0.181); (ii) odor intensity remained high for both polymers after all treatments (PP: 5.5–7.5; PE: 5–6); (iii) in PP, 30 of 32 odorants were identified versus 28 of 32 in PE; and (iv) the highest OD factors in PP (6561 for eugenol, verdyl acetate, methylcyclomyrcetone isomer) exceeded those in PE (maximum OD 2187). These comparisons are discussed in detail below, with a summary presented in Table 2.

**TABLE 2:** Side‐by‐side comparison of key sensory and analytical outcomes for post‐consumer rigid PE (present study) and PP [13]. Both polymers were sourced from the same Belgian MRF, processed under identical washing conditions, and evaluated by the same trained panel. Medians are reported; statistical significance is indicated by an asterisk (*). n.s. = not significant.

**Parameter**	**PE**	**PP [13]**
**Sample source**	HDPE rigid packaging (bottles, containers); Belgian MRF	PP copolymers (bottles); same Belgian MRF
**Trained panel**	*n* = 8 (5F/3M, 23–35 y); within‐subject	Same 8 panelists; within‐subject
**Washing conditions**	RT (25°C), HW (80°C), DW (2 m% NaOH + CTAB, 80°C)	Identical: RT, HW, DW
**Hedonic ratings (medians, 0–10)**		
UW	2	2
RT	3	4
HW	3	3.5
DW	3.5	5
*Kruskal–Wallis*	*H = 4.88, p = 0.181 (n.s.)*	H = 11.17, p = 0.011*
*Post‐hoc (UW vs DW)*	*n.s*.	p = 0.001*
**Intensity ratings (medians, 0–10)**		
UW	6	7.5
RT	5.5	6
HW	6	7
DW	5	7.5
*Kruskal–Wallis*	*H = 3.64, p = 0.303 (n.s.)*	H = 8.19, p = 0.042* (no pairwise sig.)
**Key odor attributes (medians)**		
Moldy (UW → DW)	5.5 → 4 (p = 0.054, n.s.)	5 → 3 (p = 0.066, n.s.)
Soapy (UW → DW)	2 → 3 (p = 0.320, n.s.)	3 → 6 (p = 0.003*)
**Analytical outcomes (cOEDA)**		
Odorants detected	32	32
Odorants identified	28	30
Maximum OD factor (UW)	2187	6561
Compounds at max OD	β‐ionone, methylcyclomyrcetone isomer, eugenol	verdyl acetate, methylcyclomyrcetone isomer, eugenol
Newly reported compounds	rose oxide, dihydromyrcenol, hexyl salicylate, α‐hexylcinnamaldehyde	rose oxide, dihydromyrcenol, hexyl salicylate, cis‐3‐hexenyl salicylate, α‐hexylcinnamaldehyde

The observed differences in odor profiles between PE and PP [13], and their change after washing treatments can be explained by the different chemical structures and surface physicochemical properties. PE has a higher surface free energy (25.9 mJ/m^2^) when compared to PP (20,2 mJ/m^2^) [26]. Thus, despite the non‐polarity of PE (0 mJ/m^2^) [26], this polymer can facilitate nonspecific adsorption of a VOCs through van der Waals forces. Moreover, the absence of polar functional groups on the surface of PE limits interaction with polar washing agents, thereby reducing the efficiency of deodorization. In contrast, PP has a small polar component (0.4 mJ/m^2^) [26]. This small polar contribution, although limited, can increase specificity in interactions with certain functional groups when compared to PE. For example, compounds such as cis‐3‐hexenyl salicylate (soapy, flowery) and eugenol (spicy, clove‐like) showed high OD factors in PP [13], even after washing. This selective retention of more desirable or less offensive odorants can also explain the observed improvement in hedonic perception for PP [13] following DW treatment in comparison to PE.

It is important to note that while washing improved hedonic ratings, it had a limited impact on overall odor intensity for both polymers. PE showed its highest odor intensity ratings in the UW and HW samples (Table 2), both with a median of 6, and PP remained consistently high in intensity across UW and DW conditions (Table 2, 7.5) [13], with only a moderate reduction in the RT samples. These results reinforce that while washing can shift odor character toward more acceptable attributes, it does not necessarily reduce the overall perceived intensity of odors.

A direct comparison between the odorant profiles of PE and PP [13] reveals both shared and polymer‐specific contamination patterns. In both polymers, eugenol (clove‐like), β‐ionone (flowery, violet‐like), and α‐isomethylionone (flowery) were detected among the most potent odorants, indicating common contamination sources from household and cosmetic products. However, the OD factors and their response to washing differed substantially between the two matrices. For instance, β‐ionone showed the highest OD factor (2187) in UW PE but was markedly reduced to 27 after HW treatment, whereas in PP [13], this compound maintained an OD factor of 2187 in the UW sample and only decreased to 243 after HW and DW washing. Eugenol, another highly potent odorant, exhibited an OD factor of 2187 in UW PE that dropped sharply to 9 after both HW and DW, while in PP [13] it retained an OD factor of 6561 in UW and was still 6561 after HW. These differences can be attributed to the distinct adsorption–desorption dynamics governed by polymer morphology and crystallinity. Higher crystallinity, as found in PE relative to PP, generally restricts the diffusion of small molecules through the polymer matrix, since crystalline regions act as impermeable barriers to molecular transport [27]. Consequently, odorants absorbed by PE are expected to reside primarily within the amorphous phase and near the polymer surface, rather than diffusing deeply into the bulk. This more superficial distribution could explain the markedly greater reduction in OD factors observed for PE upon hot water washing, as surface‐localized contaminants are more readily mobilized at elevated temperatures. In contrast, PP's lower degree of crystallinity may allow odorants to permeate more deeply into the amorphous bulk, making them less accessible to aqueous washing and contributing to the persistent high OD factors observed even after treatment.

Results from the sensory panel gives highlight to important differences in how washing alters the perceived odor quality of the two polymers. In PP [13], DW treatment significantly improved the hedonic rating (median from 2 to 5, p = 0.001), while in PE, the hedonic improvement was more modest (median from 2 to 3.5) and not statistically significant. Similarly, the odor intensity remained high for both polymers after all washing treatments, with PP showing median intensities of 7–7.5 across all conditions [13], while PE ranged from 5 to 6. The higher overall intensity in PP may be related to the presence of additional high‐OD compounds such as verdyl acetate (OD 6561) and methylcyclomyrcetone isomer (OD 6561), both of which were found at lower OD factors in PE (243 and 2187, respectively).

## 5 Conclusions

The main objectives of the present study were to characterize odorous contaminants and to evaluate the overall odor reduction in the polymer after different washing processes. In general, 32 compounds were detected by GC‐O in each of the plastics after washing. Identification with GC‐GC‐O/MS was achieved for 28 in rigid PE plastic, and the substances rose oxide, dihydromyrcenol, hexyl salicylate, and α‐hexylcinnamaldehyde, were reported for the first time in post‐consumer PE.

The sensory evaluations corroborated the chemoanalytical analysis, reinforcing the factual contamination of post‐consumer PE in regards to smell.

This study additionally revealed that different washing processes of post‐consumer PE did not significantly change the overall intensity of the odor. However, a qualitative change was observed in the general odor profile of the washed fractions with respect to specific sensory impressions. cOEDA and GC‐O analyses indicated reductions in OD factors for several odorants, particularly with DW and RT treatments. Based on median intensity values, DW showed a trend toward the largest reduction (approximately 17%), followed by RT (approximately 8%), while HW showed no improvement compared to the unwashed sample; however, none of these differences reached statistical significance (*n* = 8 per group), and this observation should therefore be considered a trend rather than a demonstrated outcome.

We could thus demonstrate that additional approaches for comprehensive odor reduction are essential to ensure high‐quality recyclates and to proceed toward a circular economy for recycled PE polymers. Beyond these measures one might consider embracing more advanced washing steps since we showed that the overall odor profile could change in concordance with the washing process applied. However, future research would require consideration of adsorption and desorption mechanisms of odorant compounds in relation to different washing agents to polymers such as PE and PP, and the applicability with respect to sustainability of such measures – considering availability, circularity, and toxicology of washing agents, as well as energy consumption in the course of these purification processes.

## Author Contributions

Tiago Belé: investigation, conceptualization, methodology, formal analysis, data‐curation, visualization, project administration, and writing – original draft. Martijn Roosen: conceptualization, methodology, writing – review and editing. Helene M. Loos: conceptualization, methodology, project administration, supervision, writing – review and editing. Steven de Meester: funding acquisition, supervision, and writing – review and editing. Andrea Buettner: funding acquisition, project administration, supervision, and writing – review and editing.

## Conflicts of Interest

The authors declare no conflicts of interest.

## Data Availability

The data that support the findings of this study are available from the corresponding author upon reasonable request.
